# Topographical functional correlates of interindividual differences in executive functions in young healthy twins

**DOI:** 10.1007/s00429-021-02388-4

**Published:** 2021-12-04

**Authors:** Arianna Menardi, Andrew E. Reineberg, Louisa L. Smith, Chiara Favaretto, Antonino Vallesi, Marie T. Banich, Emiliano Santarnecchi

**Affiliations:** 1 Precision Neuroscience and Neuromodulation Program, Gordon Center for Medical Imaging, Massachusetts General Hospital, Harvard Medical School, Boston, MA USA; 2 Padova Neuroscience Center & Department of Neuroscience, University of Padova, Padua, PD Italy; 3 Institute for Behavioral Genetics, University of Colorado Boulder, Boulder, CO USA; 4 Department of Psychology and Neuroscience, University of Colorado Boulder, Boulder, CO USA; 5 IRCCS San Camillo Hospital, Venice, Italy; 6 Institute of Cognitive Science, University of Colorado Boulder, Boulder, CO USA

**Keywords:** Brain topology, Executive functions, Twins study, Heritability, Graph theory

## Abstract

Executive functions (EF) are a set of higher-order cognitive abilities that enable goal-directed behavior by controlling lower-level operations. In the brain, those functions have been traditionally associated with activity in the Frontoparietal Network, but recent neuroimaging studies have challenged this view in favor of more widespread cortical involvement. In the present study, we aimed to explore whether the network that serves as critical hubs at rest, which we term network reliance, differentiate individuals as a function of their level of EF. Furthermore, we investigated whether such differences are driven by genetic as compared to environmental factors. For this purpose, resting-state functional magnetic resonance imaging data and the behavioral testing of 453 twins from the Colorado Longitudinal Twins Study were analyzed. Separate indices of EF performance were obtained according to a bifactor unity/diversity model, distinguishing between three independent components representing: Common EF, Shifting-specific and Updating-specific abilities. Through an approach of step-wise in silico network lesioning of the individual functional connectome, we show that interindividual differences in EF are associated with different dependencies on neural networks at rest. Furthermore, these patterns show evidence of mild heritability. Such findings add knowledge to the understanding of brain states at rest and their connection with human behavior, and how they might be shaped by genetic influences.

**Supplementary Information:**

The online version contains supplementary material available at 10.1007/s00429-021-02388-4.

## Introduction

Executive functions (EF) are an umbrella term for high-order cognitive abilities employed for situations requiring goal-directed behavior, including the maintenance of task goal(s), the integration and elaboration of incoming lower-level signals and processes for the selection of relevant information and inhibition of distractors, switching between concurrent goals, and overall decision making (Banich 2009). The neurological underpinnings of brain organization are commonly investigated by using fluctuations in the amount of blood (de-)oxygenation, known as Blood Oxygen Level Dependent (BOLD) signal, across distant cortical regions (Anderson et al. 2008). This approach has unveiled complex cortical profiles, whereby regions show patterns of co-activation and spontaneously assemble into coherent networks at rest, which mimic those observed during task (Smith et al. 2013). Traditionally, cognitive processes underlying EF have been thought to rely on the metabolic and functional activity of frontal and parietal regions (Stuss et al. 1998; Collette et al. 2005), which form the Frontoparietal Network (FPN). However, recent neuroimaging studies have expanded this view in favor of a more widespread involvement of remote brain regions in EF as well, including of posterior and subcortical structures (Jurado and Rosselli 2007; Fedorenko et al. 2013). This evidence suggests that interconnectedness and integrity across multiple brain regions may help sustaining executive functioning. As such, investigating inter-network organization may be a fruitful route for understanding interindividual differences in EF, especially considering the role that regions outside the FPN might play in supporting higher levels of EF.

Since EF abilities rely upon distributed brain functional patterns, great interest has been directed toward the study of interindividual differences in their organization as assessed by resting-state patterns of connectivity. Indeed, not only have interindividual differences in the functional connectome been proven to represent a unique signature of the individual (Finn et al. 2015), but they have also been proven highly stable in the adult brain (Ronnlund et al. 2015). These findings have motivated the development of new approaches to help characterize individual differences, with such approaches showing that most of the regions helpful in improving interindividual differentiation lie in the default mode, attentional and control executive networks (Airan et al. 2016). Such findings are consistent with prior studies that reveal how the network organization of both the FPN and other attentional networks, as well as their coupling, differentiate higher versus lower EF individuals (Reineberg et al. 2015, 2018).

More recently, the study of brain networks has been conceptualized within the framework of graph theory measures that allow for investigations of integration and segregation mechanisms that ensure information flow across the brain (Sporns 2013). Graph theory approaches deal with the study of network topology looking at the relationship between the graph nodes (i.e., brain regions) and edges (i.e., functional or structural connections). The study of interindividual differences has been approached with the use of several graph theory measures. These have shown evidence of a tight link between global efficiency measures and interindividual differences in intelligence scores (Li et al. 2009). Of interest, the degree of global connectivity of the prefrontal cortex has also been shown to selectively relate with interindividual differences in intelligence, suggesting a role of this region as a major hub for information exchange in the brain that is necessary for higher-order cognitive behavior (Cole et al. 2012; Duncan et al. 2020). In line with the aforementioned studies, high and low EF individuals show distinct network topologies at rest with hub-like behavior for high EF individuals occurring for some regions outside the traditional FPN (Reineberg and Banich 2016).

In the present study, we expand upon these findings in two ways. First, we used in silico lesioning of the functional connectome (Hart et al. 2016) to examine network characteristics as a function of an individual’s level of EF. In these in silico approaches, the network topology at baseline is determined. Then, networks are selectively lesioned iteratively in a step-by-step manner. In our case, in each step we took out the brain node (or edge) that is associated with the highest level of hub-like activity, referred to as centrality. In our study, centrality of nodes was determined based on their nodal degree, a measure representing the amount of connection that a given brain region has to the rest of the brain. While several other measures of centrality have also been utilized in the literature, they tend to yield similar results (Joyce et al. 2013). This approach is then run iteratively, such as that every time a node is removed from the individual connectivity matrix, centrality of all nodes is recalculated and the “new” most central node is removed. Because the most central nodes for information transfer are removed first in this procedure, one can determine those regions that play the most important role in the system across varying levels of degradation. Networks which lost the greatest number of nodes during the first stages of lesioning were considered to represent the networks on which individuals most rely during rest, which we term “network reliance”.

Lesioning approaches have a long history in network science, as they provide a window on the role of network topology in sustaining efficient information processing (Barabasi and Bonabeau 2003). For example, in silico lesioning studies of scale-free systems have revealed that efficiency comes with a trade-off in the resilience of the network to sustain the random or targeted attack of its nodes, as the removal of central hubs causes greater disruption than the removal of random nodes (Barabasi and Bonabeau 2003). Particularly efficient networks, like the brain, appear to be able to withstand both targeted and random attacks (Achard et al. 2006; Joyce et al. 2013).

The second manner in which our study extends beyond prior work is that it was performed on a set of twins (in their late 20 s) which provides the opportunity to examine whether genetic or environmental influences are most prominent in an individual’s network characteristics as a function of EF. We did so by exploring how these brain patterns of network reliance differ between pairs of monozygotic (MZ) twins, who share 100% of their genes, as compared to dizygotic (DZ) twins, who share half their genes. Twins’ studies represent indeed one of the most common approaches for the investigation of genetic influences on a trait and hence its heritability (Mayhew and Meyre 2017; Friedman et al. 2021). In recent years, twins’ studies have for instance been employed to demonstrate the genetic underpinnings of EF (Lessov-Schlaggar et al. 2007; Friedman et al. 2008; Tucker-Drob et al. 2013), the genetic influences in shaping the individual functional and structural connectome (Thompson et al. 2013; Reineberg et al. 2019), the heritability of the topological organization of the human connectome (Bolken et al. 2014; Sinclair et al. 2015), as well as the heritability of resilience to its systematic lesioning (Menardi et al. 2021). However, to our knowledge, no study has tried to combine this evidence to verify if in silico lesioning can be used to unveil patterns of preferential network reliance at rest as a function of individual EF abilities, nor if such reliance is at least partially influenced by genetic factors.

## Methods

### Participants and assessment of executive functioning

A total of 453 twins from the Colorado Longitudinal Study (age: *M* = 28.6, SD =  ± 0.62; MZ: *n* = 229, *M* = 28.6, SD =  ± 0.62; DZ: *n* = 216, *M* = 28.7, SD = 0.63) were recruited based on birth records from the Colorado Twin Registry. All participants underwent cognitive testing particularly focused on determining EF. From all tests’ scores, three EF components in the form of z-values were extracted according to the unity and diversity model by Miyake and colleagues (Miyake et al. 2000; Friedman and Miyake 2017), consisting of a common EF (cEF) factor, representative of the shared variance underlying all the administered EF tasks; and the updating (UPD) and shifting (SHI) specific components, reflecting the remaining correlation between EF tasks once the cEF factor is removed. For more details on the model, the readers are referred to the original published work (Miyake et al. 2000; Friedman and Miyake 2017).

To determine the amount of overlap in participants’ scores across EF factors, higher and lower performers for each of the three EF components (cEF, SHI, UPD) were determined based on a cut-off score around 0, such as that z-values above 0 were considered as representative of individuals whose performance was above the sample mean, thus referred as “higher performers”, whereas z-values below 0 were considered indicative of “lower performers” within the same sample. Overall, the 67% and 65% of the subjects classified as higher performers for the cEF component were also observed to show higher performance on the SHI and UPD components, respectively; on the other hand, 45% of higher SHI performers also had higher scores at the UPD component. In this study, a moderate positive correlation was observed between our measures of cEF and SHI (*r* = 0.31, *p* < 0.0001) and between cEF and UPD (*r* = 0.38, *p* < 0.0001); with a negative correlation between SHI and UPD measures (*r* = − 0.28; *p* < 0.0001).

To control for the possible risk of multicollinearity, the Variance Inflation Factor (VIF) was computed for each cognitive measure. VIF values inform on the percentage of the coefficients’ variance that is inflated, where values close to 1 indicate no correlation, values between 1 and 5 indicate moderate correlation and values above 5 suggest high correlation (Fox 2015). Our results prove a VIF near 1 for all the three EF factors (VIF_cEF_ = 1.49; VIF_SHI_ = 1.38; VIF_UPD_ = 1.46), suggesting the correlations to be only mild and that they do not imply a risk of multicollinearity of the data (Fox 2015).

### Neuroimaging data acquisition and preprocessing

All participants underwent a single scanning session in either a Siemens Tim Trio (3 T) or Prisma (3 T) scanner for the acquisition of T1 anatomical images (repetition time (TR) = 2400 ms, echo time (TE) = 2.07 ms, matrix size = 320 × 320 × 224, voxel-size = 0.80 × 0.80 × 0.80mm^3^, flip angle (FA) = 8.00 deg., slice thickness = 0.80 mm) and T2* resting-state functional magnetic resonance imaging (rs-fMRI) lasting 6.25 min (number of volumes = 816, TR = 460 ms, TE = 27.2 ms, matrix size = 82 × 82 × 56, voxel-size = 3.02 × 3.02 × 3.00mm^3^, FA = 44.0 deg., slice thickness = 3.00 mm, field of view (FOV) = 248 mm). Details on the preprocessing steps have been published in a recent work by some of the authors (Reineberg et al. 2019) and include: signal stabilization via the removal of the first 10 volumes, head motion correction, coregistration, normalization to the standard MNI152 template, denoising from motion and other noise signals through the AROMA ICA procedure and finally band-pass filtering (0.001–0.08 Hz). Blood Oxygen Level Dependent (BOLD) time series were extracted from each of the 264 spherical 1 cm parcels of the Power’s Atlas (Power et al. 2011). For each participant, a 264 × 264 functional connectivity matrix was then computed from the Pearson’s *r* Correlation between each pair of parcels and further underwent normalization through Fischer’s *z* transformation.

### In silico networks’ lesioning

To obtain a quantitative estimate over network reliance as a function of performance scores at the three EF components, we adopted a lesioning approach based on the selective removal of each node (parcel) from the original correlation matrix. In more detail, weighted adjacency matrices were obtained from the thresholded functional matrices, retaining only the 10% of the overall connection density. The rationale for such a stringent approach comes from prior large cohort studies demonstrating that 10% sparsity thresholds have the highest test–retest reproducibility for global metrics (Wang et al. 2011). Furthermore, genetic contributions have been reported to be better exploited at connection density around 10% (Sinclair et al. 2015). Nevertheless, as common in the graph theory literature, we have also tested a range of lower thresholds (from 80 to 60%), retaining from the 20% to the 40% of the original connection densities. Results comparing different thresholding are available in the Supplementary Materials, and demonstrate that the pattern of results is maintained across different thresholds.

The study of brain network properties was then approached within the graph theory framework, whereby graphs are constructed considering brain parcels as nodes and their functional correlation as weighted links, known as edges. A measure of nodal degree, an index of connectedness between a node and all the other nodes of the brain, was computed based on the Brain Connectivity Toolbox (https://sites.google.com/site/bctnet/) function running in Matlab 2017b. The measure of nodal degree was weighted based on the individual adjacency matrix, and hence it was computed as the total sum of the weighted links connecting a given node to all other nodes of the network. Nodes were then ranked based on their nodal degree, such as that parcels ranking first are thought to represent major hubs in the information flow, whereby their lesioning is expected to cause a greater disruption in network connectivity (Jeong et al. 2000), compared to the removal of less central nodes (Albert et al. 2000).

To investigate the patterns of network degradation, we progressively decomposed the functional matrix through the selective removal of one node at a time, iteratively re-computing the rank order at each stage of degradation. We then inferred the relevance of each network by counting the average number of nodes lost per network across 8 equal stages of matrix lesioning, each accounting for 33 nodes loss (i.e., equal to a 12.5% reduction in network components), totalizing 264 nodes. We hypothesized that nodes lost at the first stages of lesioning represent the brain regions and connections that are most important in network integrity as they count the most in ensuring proficient information flow. In contrast, regions and connections lost at later stages have instead a minor role in ensuring efficient functioning.

Linear regression models, accounting for dependence between twins and scanner type (TRIO, PRISMA), were then run to test the association between EF components’ scores and the associated number of nodes lost for each network, which were both treated in the form of continuous variables in the formula:

$$\mathrm{Nodes lost}\sim \mathrm{cEF}+\mathrm{SHI}+\mathrm{UPD}+\mathrm{scanner type}.$$

**Fig. 1:**
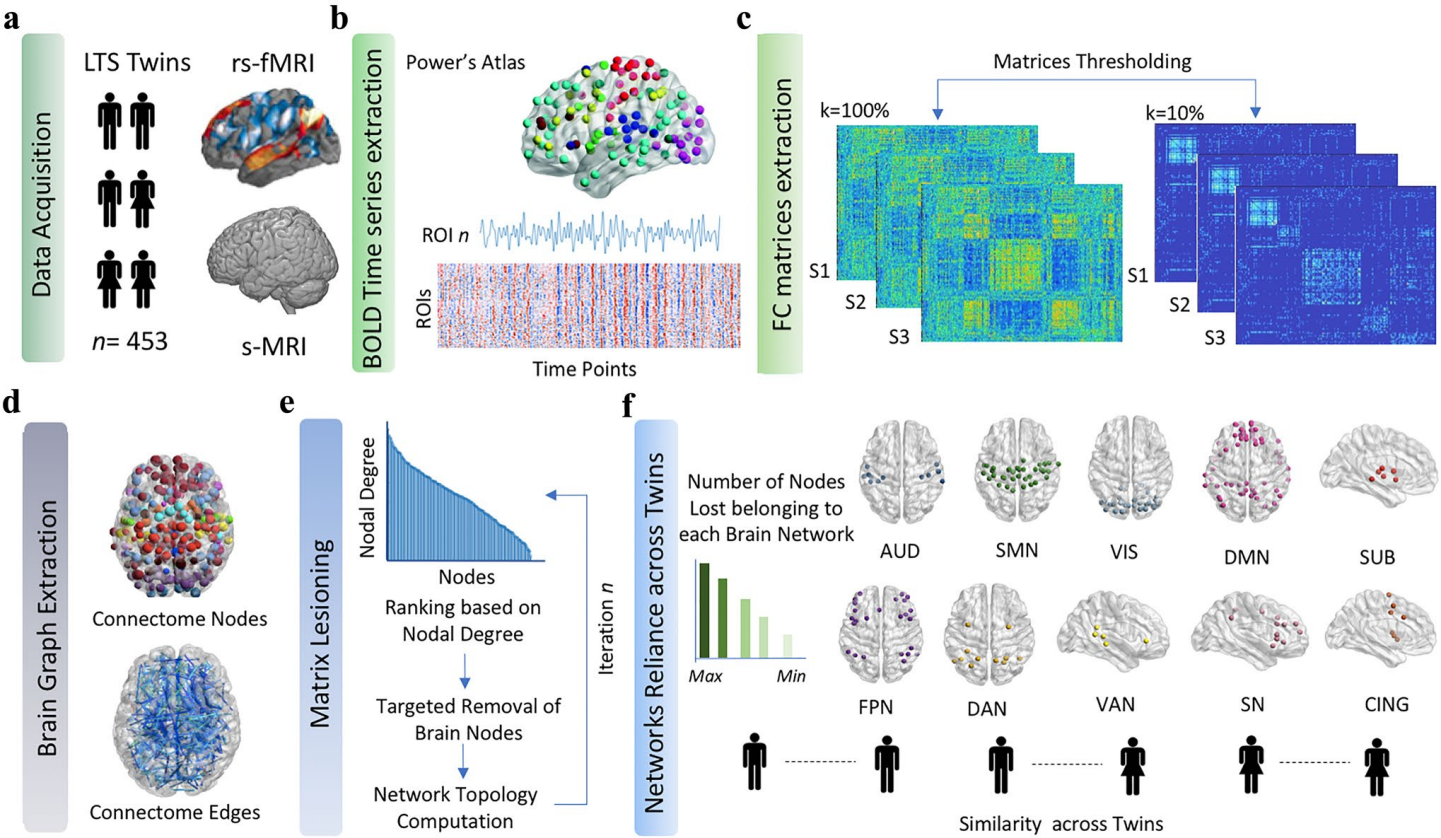
Data acquisition and analysis workflow. **a** Structural and functional MRI data were acquired from 453 twins from the Colorado LTS dataset. **b.** BOLD signal fluctuations were then extracted from each of the 264 cortical nodes as defined by the Power Atlas (Power et al. 2011) and used to extract individual functional connectivity matrices from the Pearson’s *r* correlation value between each pair of ROIs. **c** Matrices were then thresholded to retain the 10% of the connection density. **d** Brain graph metrics were extracted, considering brain parcels as nodes and their functional connections as edges. **e** A matrix lesioning approach was employed to estimate the extent for which individual brain activity at rest relies on cortical nodes belonging to different cortical networks. To do so, all nodes in the brain were ranked based on their nodal degree, so that the most important hub in the brain was removed first during the lesioning process. After each lesion, brain topology measures were re-computed and the order of lesioning updated. **f** At the end of the lesioning process, we counted the number of nodes belonging to a given network that were lost at each stage of matrix lesioning. Networks which lost the greatest number of nodes during the first stages of lesioning (reduction of 12.5% of nodes) were considered to represent the networks on which individuals most rely during rest (i.e., network reliance). Finally, the influence of genetics and of environmental factors in determining network reliance was computed as the difference between MZ and DZ twins. *AUD auditory network, *CING* cingulo-opercular network, *DAN* dorsal attention network, *DMN* default mode network, FPN fronto-parietal network, LTS Colorado Longitudinal Twin Study, *rs-fMRI* resting-state functional magnetic resonance imaging, *s-MRI* structural magnetic resonance imaging, *SMN* sensorimotor network, *SN* salience network, *SUB *subcortical network, *VAN* ventral attention network, *VIS* visual network

Significance threshold value was set as *p* = 0.05. Figure 1 graphically presents the main methodological steps underlying neuroimaging data preprocessing, definition of matrices and in silico lesioning of networks.

### Heritability analyses

Monozygotic and dizygotic twins born and raised in the same family represent a unique sample of data from which to derive estimates of a trait’s heritability. In particular, pairs of MZ twins share 100% of their genetic profile, or additive genetic variance (*A*). Moreover, they are likely to receive equal maternal care, education, and socioeconomic benefits, thus also sharing a common environment (*C*) (Mayhew and Meyre 2017). Similarly, DZ twins also share the environment in which they grow but, differently from MZ twins, they only share 50% of their genes (Mayhew and Meyre 2017). Based on these notions, Falconer’s formula (Mayhew and Meyre 2017) is commonly employed to estimate heritability of a given human trait from the correlation between MZ and DZ twins, multiplied by 2:

$${H}^{2}=2\left({r}_{mz}-{r}_{dz}\right).$$

According to this formula, if a trait presents a significantly higher correlation in MZ twins compared to DZ twins, then that trait can be considered as genetically influenced, whereas if a higher DZ correlation is observed compared to MZ, then that trait might be more driven by environmental factors instead. Although the Falconer’s formula has the advantage of providing a fast and easily computable estimate of heritability, more information can be derived from structural equation models (SEMs) (Mayhew and Meyre 2017). In genetic studies, SEMs are typically employed for the comparisons of 4 models: ACE, which considers the influence of A, C and of unique (E) environmental influences plus measurement error, of only AC (dropping E), of only AE (dropping C) and finally of only E models. This approach has the advantage of allowing an estimation of the contribution of each specific factor (A, C and E) to the phenotype. In the present study, SEMs were compute via OpenMx (Neale et al. 2016). The best model fit was chosen based on the model with a non-significant Chi-Square test and the lowest Akaike Information Criteria (AIC), as those are indicative of models with smaller prediction error (Wilson and Hilferty 1931; Akaike 1973).

In this study, Falconer’s formula was used to derive initial estimates of heritability, followed by a more throughout analysis via SEMs. The heritability of EF was assessed for replication purposes, since prior studies have already determined their genetic origin (Lessov-Schlaggar et al. 2007; Friedman et al. 2008; Tucker-Drob et al. 2013). On the other hand, a more in-depth analysis was carried out to explore the extent to which resting-state networks’ reliance, computed from the average node loss per network in the first four stages of lesioning (up to 50% of the network is lesioned), could be considered genetically influenced and, thus, heritable.

## Results

### Differential networks reliance as a function of EF performance

**Fig. 2:**
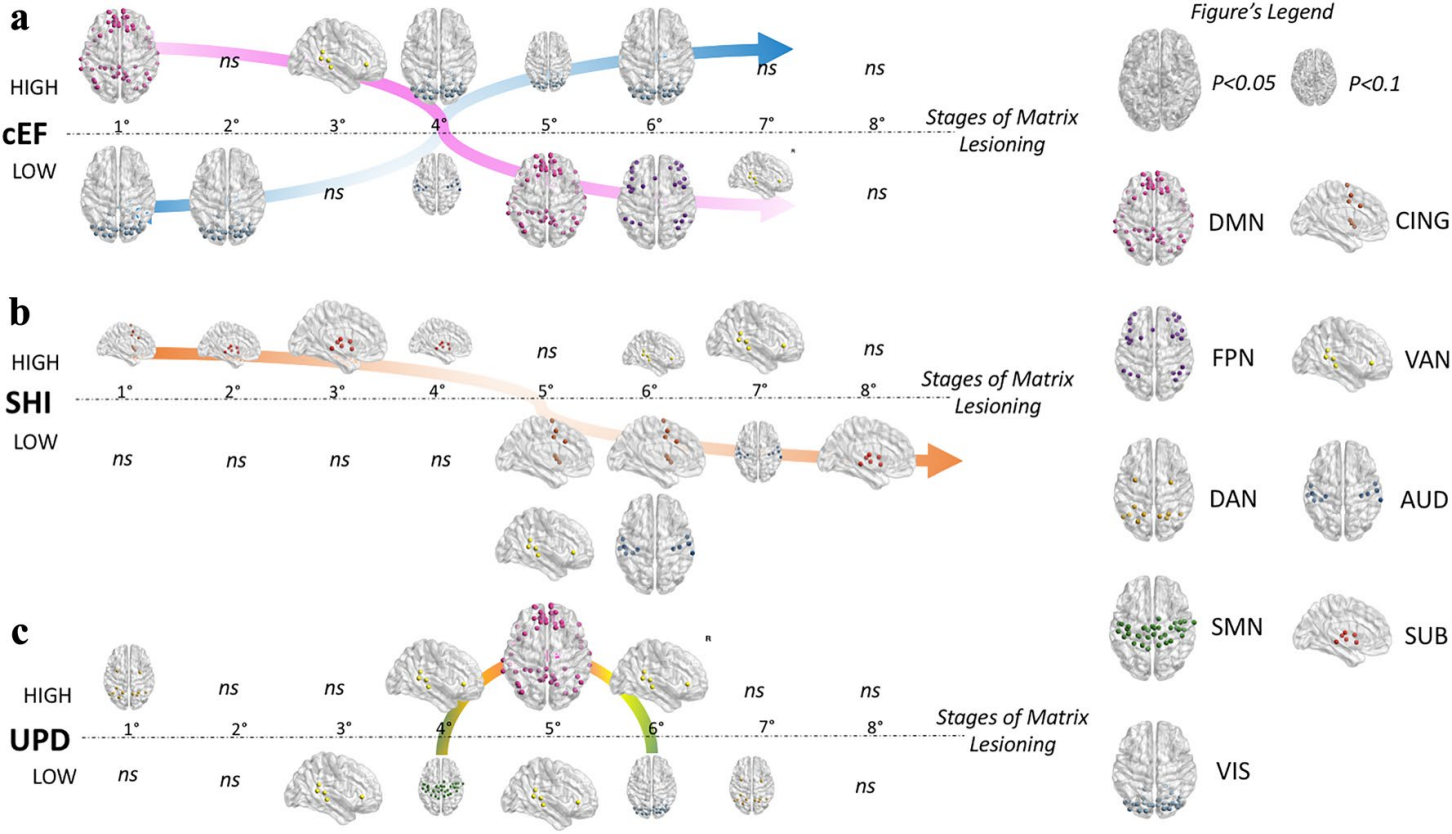
Network reliance at rest as a function of EF performance**.** Patterns of network reliance were computed based on the average number of nodes lost per network for each stage of matrix lesioning. Overall, degradation patterns appeared to differ as a function of EF performance. **a** Individuals scoring higher across EF tasks (cEF component) showed an initial greater loss of DMN nodes, opposite to the lower performers, who appeared to rely more on VIS nodes instead. Interestingly, the pattern switched along the lesioning process, with high and low performers losing visual and DMN/FPN nodes at the last stages of lesioning, respectively. **b** Performance scores for the shifting-specific (SHI) component proved that, even at rest, higher performers tend to rely more on CING and SUB network nodes, whereas those same networks appear of less relevance in low performers, who lose them only at the last stages of lesioning. **c.** Higher and lower performers at the updating-specific (UPD) component tended to equally rely more on bottom-up attentional networks (VAN), with a slight tendency for higher performers to also lose more DMN nodes. In the legend, brain size is indicative of the associated statistical significance of each finding. *AUD auditory network, *cEF* common executive function, *CING* cingulo-opercular network, *DAN* dorsal attention network, *DMN* default mode network, *FPN* fronto-parietal network, *ns* non-significant, *SHI* shifting-specific factor, *SMN* sensorimotor network, *SN* salience network, *SUB* subcortical network, *UPD* updating-specific factor, *VAN* ventral attention network, *VIS* visual network

Networks’ lesioning via the targeted removal of nodes based on their nodal degree was used to explore patterns of networks reliance as a function of EF performance. Results of linear regression models yielding a significant, positive beta value indicated that individuals with higher EF scores were losing more nodes belonging to that specific resting-state network than those with lower EF scores, while negative beta values indicated the opposite: individuals with lower EF scores were losing more nodes belonging to a specific network than those with higher EF scores. Figure 2 shows a graphical depiction of the observed patterns of network reliance as a function of EF performance. Associated raw beta and p values for each stage of matrix lesioning are listed in Table 1. In consideration of potential type I errors due to multiple comparisons, *p* values surviving false-discovery rate (FDR) correction (Jafari and Ansari-Pour 2019) are marked in bold in Table 1. For each network, FDR was applied to consider multiple testing over the 8 stages of matrix lesioning.

**Table 1:** Patterns of network reliance as a function of EF performance

		Stages of lesioning							
		1st stage	2nd stage	3rd stage	4th stage	5th stage	6th stage	7th stage	8th stage
Network reliance at rest	DMN	cEF *b* = 1.15 *p* = 0.036	–	–	–	cEF *b* = − 0.41 *p* = 0.041 UPD *b* = 0.54 *p* = 0.034	–	–	–
	FPN	–	–	–	–	–	cEF *b* = − 0.3 *p* = 0.046	–	–
	VIS	cEF *b* = − 0.91 *p* = 0.037	cEF *b* = − 0.53 *p* = 0.024	−	cEF *b* = 0.45 *p* = 0.018	**− **	cEF *b* = 0.35 *p* = 0.026	–	–
	CING	–	–	–	–	SHI *b* = − 0.23 *p* = 0.021	SHI *b* = − 0.19 *p* = 0.044	–	–
	SUB	–	–	**SHI** ***b* = 0.3** ***p* = 0.002**	–	–	**− **	–	SHI *b* = − 0.38 *p* = 0.019
	AUD	–	–	–	–	–	**SHI** ***b* = − 0.38** ***p* = 0.006**	–	–
	VAN	–	–	cEF *b* = 0.2 *p* = 0.047	UPD *b* = 0.21 *p* = 0.039	**SHI** ***b* = − 0.22** ***p* = 0.004** UPD *b* = − 0.19 *p* = 0.039	**UPD** ***b* = 0.28** ***p* = 0.004**	**SHI** ***b* = 0.2** ***p* = 0.012**	–

Multilevel models, accounting for dependence between twins and scanner type, were used to test the association between EF components’ scores and the associated number of nodes lost for each network. Associated raw beta and *p* values are reported across stages of matrix lesioning for all networks reaching the significant value (*p* < 0.05). The significant values surviving the FDR correction for multiple comparison over the 8 stages of lesioning are marked in bold. Overall, positive beta values are indicative of individuals with higher EF scores losing more nodes belonging to that specific resting-state network. The opposite, significant negative beta values, are indicative of individuals with lower EF scores losing more nodes belonging to a specific network

*AUD* auditory network, *cEF* common executive function, *CING* cingulo-opercular network, *DMN* default mode network, *FPN* fronto-parietal network, *SHI* shifting-specific factor, *SUB* subcortical network, *UPD* updating-specific factor, *VAN* ventral attention network, *VIS* visual network

For cEF, none of the results survived the correction for multiple comparisons. Nonetheless, a number of statistically significant effects were observed and they are described here for completeness. Higher scoring individuals tended to lose nodes belonging to the Default Mode Network (DMN) at the very first stage of lesioning (b_1_ = 1.15, p_1_ = 0.036), opposite to what observed in those with lower EF, who tended to lose sensory nodes belonging to the Visual (VIS) network in the first two steps of matrix lesioning (b_1_ = − 0.91, p_1_ = 0.037; b_2_ = − 0.53, p_2_ = 0.024). Interestingly, the observed pattern appeared to flip toward the final stages of matrix lesioning, with higher cEF individuals losing VIS nodes at stages 4 and 6 (b_4_ = 0.045, p_4_ = 0.018; b_6_ = 0.35, p_6_ = 0.026) and lower cEF losing DMN and FPN nodes at stage 5 and 6 (DMN: b_5_ = − 0.41, p_5_ = 0.041; FPN: b_6_ = − 0.29, p_6_ = 0.046).

With regard to the SHI component, results surviving the FDR correction showed a significant positive correlation between performance scores at SHI-specific abilities and the higher loss of nodes belonging to the Subcortical (SUB) network at stage 3 (b_3_ = 0.3, p_3_ = 0.002) and Ventral Attention (VAN) at stage 7 (b_7_ = 0.2, p_7_ = 0.012), and conversely a negative correlation with the loss of nodes belonging to the Auditory (AUD) network at stage 6 (b_6_ = − 0.38, p_6_ = 0.006) and the VAN at stage 5 (b_5_ = − 0.22, p_5_ = 0.004). Although not surviving correction for multiple comparisons, we also observed a trend in the data whereby lower SHI performers tended to show a higher loss of nodes belonging to the cingulo-opercular (CING) and SUB networks at later stages of lesioning (5th, 6th and 8th (i.e., final)) (CING: b_5_ = − 0.23, p_5_ = 0.021; b_6_ = − 0.19, p_6_ = 0.044; SUB: b_8_ = − 0.38, p_8_ = 0.019).

In regard of UPD-specific abilities, results surviving the FDR correction showed a significant higher loss of nodes belonging to the VAN at stage 6 of lesioning (*b*_6_ = 0.28, *p*_6_ = 0.004) in individuals with higher performance scores. In addition, although not surviving correction for multiple comparisons, we observed a tendency by the higher performers to also loose VAN nodes at stages 4 of matrix lesioning (b_4_ = 0.21, p_4_ = 0.039) and more DMN nodes at stage 5 (b_5_ = 0.54, p_5_ = 0.034).

### Genetic influence in neuronal networks reliance and EF performance

As found in previous waves of this study (Friedman et al. 2008; Friedman and Miyake 2017), high heritability was observed for the measures of EF performance. In general, higher correlations for EF scores were observed in MZ twins compared to DZ twins, suggesting very similar cognitive profiles across pairs of MZ twins. As a result, moderate-to-high heritability estimates were observed for the cEF factor (rMZ = 0.81, rDZ = 0.22, Falconer’s value = 1.18), followed by the SHI (rMZ = 0.55, rDZ = 0.15, Falconer’s value = 0.79) and UPD (rMZ = 0.65, rDZ = 0.32, Falconer’s value = 0.68) components (see Table 2).

**Table 2:** Heritability Estimates of EF

EF components	*rMZ*	*rDZ*	Falconer’s Value
cEF	0.81	0.22	1.18
SHI	0.55	0.15	0.79
UPD	0.65	0.31	0.68

The extent of genetic influence on EF performances was calculated by means of Falconer’s formula, computed from the correlation between MZ and DZ twins. All three EF components showed moderate-to-high genetic influences

*cEF *common executive function, *DZ *dizygotic twins, *MZ *monozygotic twins, *SHI *shifting-specific factor, *UPD *updating-specific factor

In the present work, we also asked whether the observed patterns of network reliance, as indexed by the average node loss in the first stages of lesioning (stage 1 to 4), was influenced by genetics and/or environmental factors. Said differently, we were examining whether the pattern of loss of nodes appears to show genetic influences. For this purpose, both the Falconer’s Formula and SEMs, comparing the models’ fitness considering all combinations between A, C and E components, were run. As shown in Table 3, both the Falconer’s Formula and the model selection based on non-significant Chi-Square and low AIC values reveal the presence of genetic influences for 3 networks: the DAN, the DMN and the FPN. For the DAN network, the Falconer’s Formula reveals a MZ correlation that is double that of DZ twins’ pairs (rMZ = 0.28, rDZ = 0.14, H = 0.29). The pairwise model comparison dropping A resulted in poorer model fit, as well as the model comparison dropping A and C. This suggests a significant contribution of genetic components to the phenotype, which is supported by the best model fit being the one combining the A and E components. A similar pattern was observed for the DMN, where the MZ correlation remained higher than the DZ correlation (rMZ = 0.46, rDZ = 0.38, *H* = 0.15), suggesting a genetic contribution. Again, the pairwise model comparison revealed the AE model as the one with the smallest prediction error and it was hence preferred over all other combinations. Finally, a slightly more complex scenario was observed for the FPN. As for the DAN and DMN, the correlation between MZ twin pairs was still higher than that of DZ twins (rMZ = 0.40, rDZ = 0.28, *H* = 0.23). However, a drop in model fit was observed whenever one of the three parameters (A, C or E) was removed from the model, suggesting that the best prediction could be achieved only when their combined contribution was considered.

**Table 3:** Heritability of network reliance at rest

Falconer’s formula			Structural equation models				
*rMZ*	rDZ	Falconer's H	Comparison	minus2LL	df	AIC	*p*
*AUD*							
0.21	0.19	0.04	ACE	996.79	395	206.79	NA
			AE	996.80	396	204.80	0.93
			CE	996.80	396	204.80	0.90
			**E**	**997.28**	**397**	**203.28**	**0.78**
*CING*							
0.20	0.25	− 0.11	ACE	1137.35	395	347.35	NA
			AE	1137.55	396	345.55	0.65
			**CE**	**1137.40**	**396**	**345.40**	**0.82**
			E	1141.35	397	347.35	0.13
*DAN*							
0.28	0.14	0.29	ACE	960.80	395	170.80	NA
			**AE**	**960.81**	**396**	**168.81**	**0.93**
			CE	961.08	396	169.08	0.60
			E	964.75	397	170.75	0.14
*DMN*							
0.46	0.38	0.15	ACE	1811.11	395	1021.11	NA
			**AE**	**1811.13**	**396**	**1019.13**	**0.90**
			CE	1811.30	396	1019.30	0.66
			E	1815.23	397	1021.23	0.13
*FPN*							
0.40	0.28	0.23	**ACE**	**1322.36**	**395**	**532.36**	**NA**
			AE	1327.02	396	535.02	0.03
			CE	1329.61	396	537.61	0.01
			E	1330.79	397	536.79	0.01
*SMN*							
0.15	0.20	− 0.11	ACE	1821.84	395	1031.84	NA
			AE	1822.48	396	1030.48	0.42
			CE	1823.00	396	1031.00	0.28
			**E**	**1823.54**	**397**	**1029.54**	**0.43**
*SN*							
0.23	0.22	0.01	ACE	1146.22	395	356.22	NA
			AE	1146.48	396	354.48	0.62
			CE	1146.50	396	354.50	0.60
			**E**	**1146.50**	**397**	**352.50**	**0.87**
*SUB*							
0.12	0.18	− 0.12	ACE	778.22	395	− 11.78	NA
			AE	778.22	396	− 13.78	0.94
			CE	778.22	396	− 13.78	0.97
			**E**	**778.39**	**397**	**− 15.61**	**0.92**
*VAN*							
0.16	0.19	− 0.05	ACE	726.57	395	− 63.43	NA
			AE	728.98	396	− 63.02	0.12
			CE	727.99	396	− 64.01	0.23
			**E**	**729.83**	**397**	**− 64.17**	**0.20**
*VIS*							
0.32	0.23	0.19	ACE	1696.06	395	906.06	NA
			AE	1696.82	396	904.82	0.38
			CE	1697.30	396	905.30	0.27
			**E**	**1697.62**	**397**	**903.62**	**0.46**

The extent of genetic influences on network reliance at rest was calculated by means of Falconer’s Formula and structural equation models. Model selection, marked in bold in the table, was based on non-significant Chi-Square test and the Akaike information criterion, whereby lower values are indicative of better fit

^*^AIC Akaike Information criterion, *AUD* auditory network, *CING* cingulo-opercular network, *DAN* dorsal attention network, *df* degrees of freedom, *DMN* default mode network, F*PN* fronto-parietal network, *minus2LL* minus 2 log likelihood value; *SMN* sensorimotor network, *SN* salience network, *SUB* subcortical network, *VAN* ventral attention network, *VIS* visual network

**Fig. 3:**
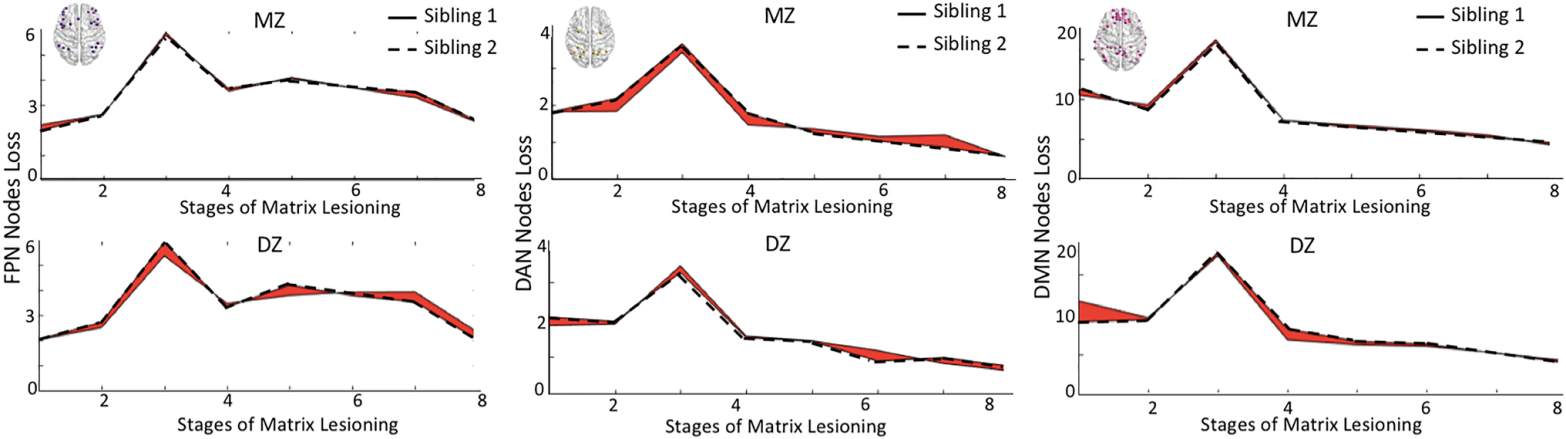
Phenotypic similarity across MZ and DZ twins**.** The average number of nodes lost for the FPN, DAN and DMN network along the 8 stages of matrix lesioning is shown. The greater the similarity in the profiles between MZ and DZ siblings, the narrower the shadowed area in-between profiles. *FPN fronto-parietal network, *DAN* dorsal attention network, *DMN* default mode network, *MZ* monozygotic, *DZ* dizygotic

Interestingly, these findings were replicated across different matrix thresholding, specifically from 80 to 60% thresholding, for both the DAN, DMN and FPN. SEMs results along all ranges of matrix thresholding are reported in Table S1 of the Supplementary Materials. The complete patterns of network lesioning for the 3 networks showing genetic influences are also displayed in Fig. 3.

## Discussion

In the present study, we expand the current knowledge of the relationship between brain and cognition by quantifying how, within a large set of neurologically normal individuals, one’s level of EF performance is associated with the differential load upon brain networks at rest. The notion that specific cortical activation patterns are observed as a function of the individual EF abilities, as assessed outside the scanner, has already been shown in the literature (see for example Reineberg et al. 2015, 2018; Reineberg and Banich 2016). These patterns of activity extend beyond the traditionally recognized frontal and parietal regions, involving subcortical and lower-level regions (Jurado and Rosselli 2007; Bettcher et al. 2016), whereby individuals with higher cognitive performance appear to benefit from more distributed resting-state network activity (Reineberg et al. 2015). An example of this diffuse processing is represented by the different network-to-network interactions that underlie individual differences in aspects of higher-level cognition, which have been reported both during resting-state conditions (Tian et al. 2013; Roye et al. 2020; Jolles et al. 2020), as well as during online task execution (Kelly et al. 2008; Sala-Llonch et al. 2012; Zhang et al. 2019).

Notably, several sources of evidence have shown that the functional patterns associated with behavior, usually detected via task fMRI, can be reliably detected via resting-state fMRI as well (Smith et al. 2013; Vidaurre et al. 2021). Indeed, recent work has unveiled this tight linkage providing the feasibility of reliably predicting interindividual differences via the mapping of resting-state components to task-evoked activity, showing the application of a wide range of machine learning tools for the prediction of behavior via rest data (Parker Jones et al. 2017; Cohen et al. 2020). Moreover, recent evidence also suggests that the capacity of resting-state data to accurately identify regions typically associated with behavior, e.g., language areas, might even be superior to what exploited via task-evoked activity (Park et al. 2020). In another study, the observed close correlation between task activity and the amplitude in fluctuation of the resting-state signal has been suggested as a possible rationale for the implementation of resting-state fMRI analysis as a possible proxy to determine task activity in non-compliant patients’ populations, where task fMRI testing is not possible (Kannurpatti et al. 2012). Taken together, all those lines of evidence support the existence of a strong relationship between networks behavior at rest and the unique profile of the individual (Finn et al. 2015).

In this study, we hypothesized that individuals would differ in their reliance on brain networks as function of their executive function ability and that this relationship would be reflected by our in silico lesioning approach. In particular, our logic was that networks for which the individuals showed higher reliance during rest and hence greater intra- and inter-network connectivity, as indexed by the graph theory metric of nodal degree, would be lost at the first stages of lesioning. For each of our cumulative measures of EF, specifically cEF, SHI and UPD, we were indeed able to observe distinguishable patterns, which we will discuss below.

For our cEF factor, we observed a tendency for a positive association between performance scores and the amount of nodes lost in the first stages of lesioning belonging to the DMN and VAN, whereas lower performance scores showed a tendency toward the preferential loss of nodes belonging to the VIS network instead. Interestingly, this pattern appeared to switch halfway through the stages of lesioning, such as that high performers lost VIS nodes only at the later stages and, vice versa: low cEF performers lost DMN, FPN and marginally VAN nodes at the last stages (Fig. 2a). However, because these findings did not survive the correction for multiple comparisons, great caution is advised in their interpretation, which we will discuss only for completeness and future reference. Indeed, the plausibility of the existence of discernable patterns as a function of performance abilities, has already been addressed (Reineberg et al. 2018). In this regard, prior studies have demonstrated that the DMN is involved not only in internal processes such as memory retrieval or mind wandering (Buckner and Carroll 2007; Hongkeun 2010; Spreng et al. 2010; Sestieri et al. 2011), but it also actively cooperates with other networks in sustaining high-order cognitive processes (Elton and Gao 2015), useful for the formation of chains of thoughts (Spreng et al. 2010) and in supporting goal-directed behavior (Smallwood et al. 2012). In line with this interpretation, greater DMN activity at rest is associated with better task performance (for a review see Anticevic and colleagues (Anticevic et al. 2012)). In contrast, the more the DMN activity is suppressed during a task, the more the activity of visual and sensory areas tends to emerge (Greicius and Menon 2004), such as that the greater the overall decoupling between VIS and DMN nodes, the better the performance at cognitive tasks, such as memory recall (Zhang et al. 2019). On the other hand, reduced activation of the DMN in favor of abnormal activity in the VIS network has been associated with important attentional deficits, as those observed in individuals suffering from Attention Deficit Hyperactivity Disorder (ADHD) (Hale et al. 2014). Taken together, we speculate that lower cEF performers might be more driven by external sensory inputs resulting in greater functional connectivity at rest between VIS nodes.

Differently from what was observed for the cEF component, shifting-specific scores, after correcting for multiple comparisons, were associated with differences between high and low performers mainly with regards to the involvement of networks mediating attentional processes and bottom-up/automatized behavior. In particular, we observed that individuals with higher SHI scores showed greater reliance on the SUB network (whose nodes were lost at the beginning of the lesioning process) and less reliance on the VAN (nodes were lost only at the last stages of lesioning), whereas individuals with lower SHI scores lost both AUD and VAN nodes in the last stages of lesioning. In addition, we also observed a tendency (that did not survive multiple comparisons) toward the loss of CING and SUB nodes in lower performers at the last stages, suggesting a less central role of those networks at rest (Fig. 2b). In line with this observation, studies on both healthy and pathological populations have reported a link between subcortical structures, especially the basal ganglia and the thalamus, and an individual’s capacity to proficiently switch one’s attentional focus (Ravizza and Ivry 2001; Green et al. 2017). Similarly, the connectivity of the anterior cingulate cortex with the prefrontal cortex is thought to help sustain high-order cognitive processes by enabling a transition from”thoughts to actions” (Paus 2001). On the other hand, VAN activity mediates the ability to attend to relevant sensory stimuli, particularly relevant in attentional shifting (Corbetta et al. 2008). Finally, the CING network has also been proposed to mediate alertness levels in the absence of predictive stimuli, such as that the greater its pre-stimulus activity, the faster the response times upon stimulus presentation (Coste and Kleinschmidt 2016). As such, it may be that greater connectivity at rest in nodes mediating attentional-relevant processes may allow for an individual to promptly respond to, and to switch between, different stimuli.

Finally, we investigated whether differences in network reliance could also be observed for the updating-specific component of the unity-diversity model (Friedman and Miyake 2017). In this case, UPD performance scores were mostly related to nodes belonging to the VAN network, when associations were corrected for multiple comparisons. This pattern was present in form of a significant positive correlation between the number of VAN nodes lost and the individual performance scores, which might suggest that higher UPD performers might benefit from greater network reliance upon this network, whose activity is relevant in mediating contextual updating and modulation of top-down processes (Geng and Vossel 2013). In addition, although not surviving correction for multiple comparisons, we also observed that individuals with higher scores tended to present greater involvement of the DMN network at the central stages of lesioning (Fig. 2c). A possible link between higher UPD abilities and the activity of the DMN is represented by the reported positive association between the extent of DMN network engagement and the greater levels of thought’s details during a working memory task (Sormaz et al. 2018; Turnbull et al. 2019).

A second aim of the present study was to address if the observed interindividual differences in brain and behavior might be explained by genetic influences, as exploited via Falconer’s Formula and SEMs’ pairwise comparison. SEMs were run to compare the efficacy of 4 models, iteratively considering all possible combinations between the A, C and E factors to respectively determine the influence of genetic, common and unique environmental influences on the trait of interest. Our results suggest the presence of genetic influences in the lesioning patterns of the DAN, DMN and FPN networks across a range of matrix thresholding scenarios. Interestingly, all three networks showing genetic influences are well-established cognitively relevant networks, with a strong role in determining EF abilities, as discussed above. Furthermore, we were able to replicate prior evidences of the strong genetic origin of EF abilities via the simpler implementation of the Falconer’s Formula, used to compare MZ and DZ measures based on the assumption that higher correlation between MZ twins, compared to DZ twins, is indicative of a genetic contribution to the measure at hand. It is, however, important to consider that the estimates made by the model on the role of additive genetic and environmental components on a given trait are specific to the population being tested (Mayhew and Meyre 2017). As a result, if any alteration is present in one of the component (genetic or environmental) as a result of sampling, this will be reflected in the model’s results as well (Mayhew and Meyre 2017).

Prior work was able to detail the complex heterogeneity of genetic influences in the brain, and in particular how it correlates with the resting-state networks (Richiardi et al. 2015), proving higher heritability for within, rather than between, regions in the brain serving similar functional roles (Reineberg et al. 2019). It is worth mentioning that prior studies have highlighted the issue that heritability estimates might be more difficult to exploit in the absence of long scan times (Menardi et al. 2021). Because this is a common issue in neuroimaging studies, a recent investigation has suggested a possible way to overcome this via the combination of task and rest fMRI data, to obtain a measure known as “general functional connectivity” (GFC), which has shown to entail stronger test–retest reliability and to display higher heritability estimates compared to when only rest or task fMRI data are analyzed (Elliott et al. 2019). It would be of interest for future studies to apply in silico lesioning considering GFC measures.

## Limitations

Overall, our study is not free of limitations. First of all, one might argue that the majority of the cited evidence in favor of a given network preponderance in explaining cognitive differences comes from studies looking at task fMRI data, whereas all our inferences come purely from rs-fMRI. As such, it might be challenging to assume that interindividual differences at rest could still hold any value in explaining task-related behavior. Nevertheless, a recent study investigating the replicability and reliability of fMRI data on a large dataset was able to prove how interindividual differences in the functional connectome are persistent across resting state and 7 different task-related scan acquisitions, spanning EF, motor, social and emotional tasks (Shah et al. 2016). This evidence supports that what is observed during the resting state is indeed characteristic of individual differences that shape intra and inter-network connections, which ultimately determine behavior. Of course, not all the brain areas show such heterogeneous profiles across individuals, but all the regions that do predict cognitive differences belong to areas of the brain with high variability (Mueller et al. 2013).

A second limitation of the present study is that our approach allows us to discern reliance on specific networks as a function of EF performance but not within regions of the same network, and also not with regards to hemispheric differences. For instance, prior studies have proven the existence of a right-hemispheric bias during selective attention tasks (Shulman et al. 2010), and a left hemispheric bias for task switching performance (Kim et al. 2012; Ambrosini and Vallesi 2016). One possibility is that our approach applied to resting-state data might present sufficient sensitivity, but diminished specificity, to detect the intrinsic and finer grade linkages of behavior to network reliance, which might otherwise be more easily examined via task-evoked activity instead.

Finally, while the high interindividual variability across our participants has allowed us to detect interesting differences in the brain–behavior continuum as a function of their cognitive performance, on the other hand it might have diminished the statistical strength of some of our findings. In particular, only some of the results survived FDR correction. Nevertheless, we believe that providing the reader with all our findings can help point to meaningful patterns, which would have otherwise been obscured by using more stringent corrections. Nonetheless, it would be advisable to attempt to replicate our findings in future studies.

## Conclusions

The present study adds knowledge on the relationship between the individual organization of the functional connectome at rest and interindividual differences in EF. In particular, we show that discernable patterns of preferential network reliance can be demonstrated as a function of EF performance, as revealed through an in silico lesioning approach of the functional connectome. Furthermore, while interindividual differences in EF showed high heritability estimates in our sample, replicating prior findings in the literature, heritability estimates of reliance on specific rs-fMRI networks as a function of level of EF were more modest.

## Supplementary Information

Below is the link to the electronic supplementary material.

Supplementary file1 (DOCX 49 kb)

## Data Availability

Data release is handled on a case-by-case basis after contacting the Authors.

## Code availability

The code used for the computation of the Nodal Degree measure is freely available from the Brain Connectivity Toolbox (https://sites.google.com/site/bctnet/).
