# Supplementary material for: Topographical functional correlates of interindividual differences in executive functions in young healthy twins
Source: Brain Struct Funct. 2021 Dec 4;227(1):49–62. doi: 10.1007/s00429-021-02388-4 (PMC8741656; doi:10.1007/s00429-021-02388-4)
Supplement: Supplementary file 1 — Supplementary file1 (DOCX 49 kb) [file 429_2021_2388_MOESM1_ESM.docx]

**Topographical functional correlates of interindividual differences in executive functions in young healthy twins**

Menardi, A.^1,2^, Reineberg, A.E.^3^, Smith, L.L.^4^, Favaretto, C.^2^, Vallesi, A.^2,5^,

Banich, M.T.^4,6†^, Santarnecchi, E.^1^ ^†*^

1. Berenson-Allen Center for Non-invasive Brain Stimulation, Beth Israel Deaconess Medical Center, Harvard Medical School, Boston, MA, USA
2. Department of Neuroscience & Padova Neuroscience Center, University of Padova, PD, Italy
3. Institute for Behavioral Genetics, University of Colorado Boulder, Boulder, CO USA
4. Department of Psychology and Neuroscience, University of Colorado Boulder, Boulder, CO, USA
5. IRCCS San Camillo Hospital, Venice, Italy
6. Institute of Cognitive Science University of Colorado Boulder, Boulder, CO, USA

**Supplementary Tables:** 1

**†** Contributed equally

***Corresponding author:**

Emiliano Santarnecchi

Berenson-Allen Center for Noninvasive Brain Stimulation, Beth Israel Deaconess Medical Center, Harvard Medical Chool, Boston, MA, UA

office +1-617-667-0326

mobile +1-617-516-9516

[esantarn@bidmc.harvard.edu](mailto:esantarn@bidmc.harvard.edu)

**Keywords:** Brain Topology, Executive Functions, Twins Study, Heritability, Graph Theory

**Acknowledgements**

We thank Dr. Naomi P. Friedman, PI, of NIH grant R01 MH063207, who shared the data set analyzed in this study as well as NIH grants R01 MH063207 and R01 AG046938 that supported the data collection.

**Matrix** **Thresholding**

In graph theory, graphs are usually constructed from the individual thresholded adjacency matrix, in which each entry represents an edge connecting two brain regions. Thresholding is applied with the intent of better exploiting topological properties of relatively spars networks, as the human brain, as well as of reducing the risk of spurious correlations in the matrix (Achard and Bullmore 2007). However, because no a priori knowledge can be derived on the best threshold to use, a range of connection densities is usually tested. In the present study, we tested SEMs results over a range of threshold between 90%-60%, thus retaining the 10-40% of connection densities (see Table S1).

| **Falconer’s Formula** | | | **Structural Equation Models** | | | | |
| --- | --- | --- | --- | --- | --- | --- | --- |
| 80% Threshold | | | | | | | |
| AUD | | | | | | | |
| rMZ | rDZ | Falconer's H | comparison | minus2LL | df | AIC | p |
| 0.06 | 0.06 | 0.00 | ACE | 800.05 | 395 | 10.05 | NA |
|  |  |  | AE | 800.05 | 396 | 8.05 | 0.97 |
|  |  |  | CE | 800.11 | 396 | 8.11 | 0.81 |
|  |  |  | **E** | **800.51** | **397** | **6.51** | **0.79** |
| CING | | | | | | | |
| rMZ | rDZ | Falconer's H | comparison | minus2LL | df | AIC | p |
| 0.08 | 0.12 | -0.08 | ACE | 923.94 | 395 | 133.94 | NA |
|  |  |  | AE | 924.43 | 396 | 132.43 | 0.48 |
|  |  |  | **CE** | **923.94** | **396** | **131.94** | **0.94** |
|  |  |  | E | 927.48 | 397 | 133.48 | 0.17 |
| DAN | | | | | | | |
| rMZ | rDZ | Falconer's H | comparison | minus2LL | df | AIC | p |
| 0.04 | 0.03 | 0.02 | ACE | 729.88 | 395 | -60.12 | NA |
|  |  |  | **AE** | **730.34** | **396** | **-61.66** | **0.50** |
|  |  |  | CE | 731.86 | 396 | -60.14 | 0.16 |
|  |  |  | E | 736.58 | 397 | -57.42 | 0.04 |
| DMN | | | | | | | |
| rMZ | rDZ | Falconer's H | comparison | minus2LL | df | AIC | p |
| 0.21 | 0.06 | 0.29 | ACE | 1697.54 | 395 | 907.54 | NA |
|  |  |  | **AE** | **1697.78** | **396** | **905.78** | **0.63** |
|  |  |  | CE | 1698.54 | 396 | 906.54 | 0.32 |
|  |  |  | E | 1702.05 | 397 | 908.05 | 0.11 |
| FPN | | | | | | | |
| rMZ | rDZ | Falconer's H | comparison | minus2LL | df | AIC | p |
| 0.11 | 0.08 | 0.05 | ACE | 1109.93 | 395 | 319.93 | NA |
|  |  |  | **AE** | **1110.16** | **396** | **318.16** | **0.63** |
|  |  |  | CE | 1111.20 | 396 | 319.20 | 0.26 |
|  |  |  | E | 1114.22 | 397 | 320.22 | 0.12 |
| SMN | | | | | | | |
| rMZ | rDZ | Falconer's H | comparison | minus2LL | df | AIC | p |
| -0.01 | 0.10 | -0.23 | ACE | 1627.47 | 395 | 837.47 | NA |
|  |  |  | AE | 1627.76 | 396 | 835.76 | 0.59 |
|  |  |  | CE | 1628.11 | 396 | 836.11 | 0.42 |
|  |  |  | **E** | **1628.63** | **397** | **834.63** | **0.56** |
| SN | | | | | | | |
| rMZ | rDZ | Falconer's H | comparison | minus2LL | df | AIC | p |
| 0.07 | 0.01 | 0.12 | ACE | 935.24 | 395 | 145.24 | NA |
|  |  |  | AE | 935.38 | 396 | 143.38 | 0.71 |
|  |  |  | CE | 935.26 | 396 | 143.26 | 0.88 |
|  |  |  | **E** | **935.74** | **397** | **141.74** | **0.78** |
| SUB | | | | | | | |
| rMZ | rDZ | Falconer's H | comparison | minus2LL | df | AIC | p |
| 0.07 | 0.09 | -0.04 | ACE | 673.73 | 395 | -116.27 | NA |
|  |  |  | AE | 674.09 | 396 | -117.91 | 0.54 |
|  |  |  | CE | 674.11 | 396 | -117.89 | 0.54 |
|  |  |  | **E** | **674.11** | **397** | **-119.89** | **0.82** |
| VAN | | | | | | | |
| rMZ | rDZ | Falconer's H | comparison | minus2LL | df | AIC | p |
| 0.02 | 0.07 | -0.10 | ACE | 524.18 | 395 | -265.82 | NA |
|  |  |  | AE | 524.47 | 396 | -267.53 | 0.59 |
|  |  |  | CE | 524.27 | 396 | -267.73 | 0.76 |
|  |  |  | **E** | **524.92** | **397** | **-269.08** | **0.69** |
| VIS | | | | | | | |
| rMZ | rDZ | Falconer's H | comparison | minus2LL | df | AIC | p |
| 0.09 | 0.02 | 0.14 | ACE | 1489.74 | 395 | 699.74 | NA |
|  |  |  | AE | 1489.74 | 396 | 697.74 | 0.95 |
|  |  |  | CE | 1489.84 | 396 | 697.84 | 0.75 |
|  |  |  | **E** | **1491.37** | **397** | **697.37** | **0.44** |
| 70% Threshold | | | | | | | |
| AUD | | | | | | | |
| rMZ | rDZ | Falconer's H | comparison | minus2LL | df | AIC | p |
| 0.05 | 0.09 | -0.09 | ACE | 786.00 | 395 | -4.00 | NA |
|  |  |  | AE | 786.09 | 396 | -5.91 | 0.75 |
|  |  |  | CE | 786.41 | 396 | -5.59 | 0.52 |
|  |  |  | **E** | **787.29** | **397** | **-6.71** | **0.52** |
| CING | | | | | | | |
| rMZ | rDZ | Falconer's H | comparison | minus2LL | df | AIC | p |
| 0.09 | 0.10 | -0.02 | ACE | 939.80 | 395 | 149.80 | NA |
|  |  |  | AE | 940.70 | 396 | 148.70 | 0.34 |
|  |  |  | **CE** | **939.84** | **396** | **147.84** | **0.84** |
|  |  |  | E | 944.81 | 397 | 150.81 | 0.08 |
| DAN | | | | | | | |
| rMZ | rDZ | Falconer's H | comparison | minus2LL | df | AIC | p |
| 0.09 | 0.01 | 0.14 | ACE | 773.78 | 395 | -16.22 | NA |
|  |  |  | **AE** | **774.74** | **396** | **-17.26** | **0.33** |
|  |  |  | CE | 776.32 | 396 | -15.68 | 0.11 |
|  |  |  | E | 779.70 | 397 | -14.30 | 0.05 |
| DMN | | | | | | | |
| rMZ | rDZ | Falconer's H | comparison | minus2LL | df | AIC | p |
| 0.18 | 0.07 | 0.22 | ACE | 1761.18 | 395 | 971.18 | NA |
|  |  |  | **AE** | **1761.19** | **396** | **969.19** | **0.93** |
|  |  |  | CE | 1761.68 | 396 | 969.68 | 0.48 |
|  |  |  | E | 1766.79 | 397 | 972.79 | 0.06 |
| FPN | | | | | | | |
| rMZ | rDZ | Falconer's H | comparison | minus2LL | df | AIC | p |
| 0.17 | 0.01 | 0.33 | ACE | 1146.20 | 395 | 356.20 | NA |
|  |  |  | **AE** | **1147.05** | **396** | **355.05** | **0.36** |
|  |  |  | CE | 1148.67 | 396 | 356.67 | 0.12 |
|  |  |  | E | 1151.46 | 397 | 357.46 | 0.07 |
| SMN | | | | | | | |
| rMZ | rDZ | Falconer's H | comparison | minus2LL | df | AIC | p |
| -0.01 | 0.07 | -0.17 | ACE | 1635.36 | 395 | 845.36 | NA |
|  |  |  | AE | 1635.40 | 396 | 843.40 | 0.83 |
|  |  |  | CE | 1635.58 | 396 | 843.58 | 0.64 |
|  |  |  | **E** | **1636.17** | **397** | **842.17** | **0.67** |
| SN | | | | | | | |
| rMZ | rDZ | Falconer's H | comparison | minus2LL | df | AIC | p |
| 0.02 | -0.01 | 0.06 | ACE | 982.81 | 395 | 192.81 | NA |
|  |  |  | AE | 983.65 | 396 | 191.65 | 0.36 |
|  |  |  | CE | 983.52 | 396 | 191.52 | 0.40 |
|  |  |  | **E** | **983.66** | **397** | **189.66** | **0.65** |
| SUB | | | | | | | |
| rMZ | rDZ | Falconer's H | comparison | minus2LL | df | AIC | p |
| -0.01 | 0.13 | -0.28 | ACE | 728.21 | 395 | -61.79 | NA |
|  |  |  | AE | 728.64 | 396 | -63.36 | 0.51 |
|  |  |  | CE | 728.56 | 396 | -63.44 | 0.55 |
|  |  |  | **E** | **728.65** | **397** | **-65.35** | **0.80** |
| VAN | | | | | | | |
| rMZ | rDZ | Falconer's H | comparison | minus2LL | df | AIC | p |
| 0.03 | 0.05 | -0.04 | ACE | 530.03 | 395 | -259.97 | NA |
|  |  |  | AE | 530.64 | 396 | -261.36 | 0.43 |
|  |  |  | CE | 530.40 | 396 | -261.60 | 0.54 |
|  |  |  | **E** | **530.83** | **397** | **-263.17** | **0.67** |
| VIS | | | | | | | |
| rMZ | rDZ | Falconer's H | comparison | minus2LL | df | AIC | p |
| 0.08 | -0.03 | 0.21 | ACE | 1512.65 | 395 | 722.65 | NA |
|  |  |  | **AE** | **1512.71** | **396** | **720.71** | **0.79** |
|  |  |  | CE | 1513.38 | 396 | 721.38 | 0.39 |
|  |  |  | E | 1517.33 | 397 | 723.33 | 0.10 |
| 60% Threshold | | | | | | | |
| AUD | | | | | | | |
| rMZ | rDZ | Falconer's H | comparison | minus2LL | df | AIC | p |
| 0.06 | 0.05 | 0.01 | ACE | 801.15 | 395 | 11.15 | NA |
|  |  |  | AE | 801.90 | 396 | 9.90 | 0.39 |
|  |  |  | CE | 802.63 | 396 | 10.63 | 0.22 |
|  |  |  | **E** | **803.48** | **397** | **9.48** | **0.31** |
| CING | | | | | | | |
| rMZ | rDZ | Falconer's H | comparison | minus2LL | df | AIC | p |
| 0.07 | 0.08 | -0.03 | ACE | 953.38 | 395 | 163.38 | NA |
|  |  |  | AE | 954.20 | 396 | 162.20 | 0.37 |
|  |  |  | **CE** | **953.50** | **396** | **161.50** | **0.72** |
|  |  |  | E | 956.38 | 397 | 162.38 | 0.22 |
| DAN | | | | | | | |
| rMZ | rDZ | Falconer's H | comparison | minus2LL | df | AIC | p |
| 0.06 | 0.04 | 0.04 | ACE | 806.06 | 395 | 16.06 | NA |
|  |  |  | **AE** | **806.80** | **396** | **14.80** | **0.39** |
|  |  |  | CE | 807.74 | 396 | 15.74 | 0.20 |
|  |  |  | E | 809.65 | 397 | 15.65 | 0.17 |
| DMN | | | | | | | |
| rMZ | rDZ | Falconer's H | comparison | minus2LL | df | AIC | p |
| 0.17 | 0.08 | 0.18 | ACE | 1792.64 | 395 | 1002.64 | NA |
|  |  |  | **AE** | **1792.71** | **396** | **1000.71** | **0.79** |
|  |  |  | CE | 1793.63 | 396 | 1001.63 | 0.32 |
|  |  |  | E | 1799.84 | 397 | 1005.84 | 0.03 |
| FPN | | | | | | | |
| rMZ | rDZ | Falconer's H | comparison | minus2LL | df | AIC | p |
| 0.12 | 0.01 | 0.22 | ACE | 1154.57 | 395 | 364.57 | NA |
|  |  |  | **AE** | **1155.66** | **396** | **363.66** | **0.30** |
|  |  |  | CE | 1157.66 | 396 | 365.66 | 0.08 |
|  |  |  | E | 1160.94 | 397 | 366.94 | 0.04 |
| SMN | | | | | | | |
| rMZ | rDZ | Falconer's H | comparison | minus2LL | df | AIC | p |
| 0.01 | 0.05 | -0.10 | ACE | 1635.91 | 395 | 845.91 | NA |
|  |  |  | AE | 1635.93 | 396 | 843.93 | 0.89 |
|  |  |  | CE | 1636.11 | 396 | 844.11 | 0.65 |
|  |  |  | **E** | **1637.01** | **397** | **843.01** | **0.58** |
| SN | | | | | | | |
| rMZ | rDZ | Falconer's H | comparison | minus2LL | df | AIC | p |
| 0.01 | -0.03 | 0.07 | ACE | 999.97 | 395 | 209.97 | NA |
|  |  |  | AE | 1000.85 | 396 | 208.85 | 0.35 |
|  |  |  | CE | 1001.20 | 396 | 209.20 | 0.27 |
|  |  |  | **E** | **1001.34** | **397** | **207.34** | **0.50** |
| SUB | | | | | | | |
| rMZ | rDZ | Falconer's H | comparison | minus2LL | df | AIC | p |
| 0.04 | 0.15 | -0.23 | ACE | 764.32 | 395 | -25.68 | NA |
|  |  |  | AE | 765.52 | 396 | -26.48 | 0.27 |
|  |  |  | **CE** | **764.78** | **396** | **-27.22** | **0.50** |
|  |  |  | E | 767.13 | 397 | -26.87 | 0.25 |
| VAN | | | | | | | |
| rMZ | rDZ | Falconer's H | comparison | minus2LL | df | AIC | p |
| 0.05 | 0.06 | -0.02 | ACE | 534.01 | 395 | -255.99 | NA |
|  |  |  | AE | 534.48 | 396 | -257.52 | 0.49 |
|  |  |  | CE | 534.28 | 396 | -257.72 | 0.60 |
|  |  |  | **E** | **534.68** | **397** | **-259.32** | **0.71** |
| VIS | | | | | | | |
| rMZ | rDZ | Falconer's H | comparison | minus2LL | df | AIC | p |
| 0.06 | -0.03 | 0.19 | ACE | 1210.13 | 395 | 420.13 | NA |
|  |  |  | **AE** | **1210.19** | **396** | **418.19** | **0.81** |
|  |  |  | CE | 1210.78 | 396 | 418.78 | 0.42 |
|  |  |  | E | 1214.59 | 397 | 420.59 | 0.11 |

**Table S1. Structural Equation models across thresholds**. The extent of genetic influences on network reliance at rest was calculated by means of Falconer’s Formula and structural equation models, comparing the goodness of fit of 4 models iteratively considering all possible combinations between the A. C and E factors. Models selection, marked in bold in the table, was based on non-significant Chi Square test and the Akaike information criterion, whereby lower values are indicative of better fit.

*AIC= Akaike Information criterion, AUD= auditory network, CING= cingulo-opercular network, DAN= dorsal attention network, df= degrees of freedom, DMN= default mode network, FPN= fronto-parietal network, minus2LL= minus 2 log likelihood value; SMN= sensorimotor network, SN= salience network, SUB= subcortical network, VAN= ventral attention network, VIS= visual network.

**References**

Achard S, Bullmore E (2007) Efficiency and Cost of Economical Brain Functional Networks. PLOS Computational Biology 3:e17. https://doi.org/10.1371/journal.pcbi.0030017
